# Improving survivability from blast injury: ‘shifting the goalposts’ and the need for interdisciplinary research

**DOI:** 10.1136/jramc-2018-000968

**Published:** 2018-05-16

**Authors:** A Phill Pearce, Jon Clasper

**Affiliations:** 1 The Royal British Legion Centre for Blast Injury Studies, Department of Bioengineering,, Imperial College London, London, UK; 2 Academic Department of Military Surgery and Trauma, Royal Centre for Defence Medicine, Birmingham, UK; 3 Defence Medical Group South East, Frimley Park, Frimley, UK

**Keywords:** survivability, blast, interdisciplinary

Blast injury is not a new phenomenon, but the nature of warfare has changed; explosive weapons are now the most common mode of battlefield trauma.^1^ In this issue, McGuire *et al* demonstrate the incidence of explosive injury in both recent UK operations and those from decades before.^2^ The need for further understanding of these injuries is apparent and this issue highlights the breadth and depth of blast injury research.

Advances have been led from the front by the Defence Medical Services. Improvements in prehospital care logistics (such as the Medical Emergency Response Team), advanced resuscitation techniques and hard fought expertise led to hitherto unseen levels of survival of severely injured personnel.^3 4^

Improved medical care is perhaps best evidenced by the cohort of ‘unexpected survivors’.^5^ These personnel sustained injuries which would not have been survivable in previous conflicts. Likelihood of survival due to any injury pattern can be predicted by a variety of scoring tools. Survival of casualties despite them sustaining a mathematically unsurvivable injury burden, while illustrating the performance of our deployed trauma system should also provoke important discussion as to the relevance of our scoring systems. Should our goalposts be shifted so that the unexpected survivor now falls within the expected category? It could be argued that scoring systems will always have to change, until we reach the point where no further medical advances are possible and the goalposts are fixed.

The UK Defence Medical Services acknowledge the limitations of injury scoring systems and, as a result, have combined them with expert opinion to highlight both paradigms and deficits in clinical care or systems. Existing scoring systems were used to stratify casualties before an expert panel determined the survivability of both ‘unexpected survivors’ and deaths.^5 6^ Review of combat-related deaths focused on those deemed to have sustained at least potentially survivable injuries. Of all deaths in this group (UK deployed forces 2002–2013), only 8% were deemed to be at least potentially salvageable.

If we do shift the score goalposts and unexpected survivors cross the threshold into the expected category, how do we continue to push for excellence? How do we determine what the next level of unexpected survivors might be when our current scoring systems are already ‘maxed out’? Discussion of those factors which may influence unexpected death and unexpected survivors is important but those expected deaths, those thought to be unsalvageable, should also be explored. Our commonly used scoring systems predict probability of death but do not discriminate or stratify level of injury beyond that which is likely to be fatal.

Such is the high performance of our deployed medical teams, it is possible that future improved survival from blast trauma can only be achieved by mitigation and prevention. This issue highlights the importance of interdisciplinary collaboration in driving forward high-quality research and translating this to clinically affect both on and off the battlefield.

Steps to further define the injury burden of potentially ‘unexpected survivors’ have been taken by examination of fatalities within particular blast scenarios.^7 8^ Two such papers are part of this issue. Webster *et al* compares pelvic injury mechanisms from the mounted and dismounted environment with suggestion of different mitigation for each.^9^ The paper by Stewart *et al* illustrates the process with pragmatic analysis of fatal head and neck injuries following under-body blast and suggests the future focus of preventative research (protection from direct head impact rather than cervical spine injury).^10^

Mitigation of injury requires, first, a detailed understanding of the mechanism by which each injury occurs and, second, quantification of the likelihood of injury in response to a specified ‘dose’ of the injurious stimulus. Mitigating and preventative measures can be designed and optimised to best dampen the relationship between dose and response. This approach is applied by the injury biomechanics community to causes of intentional and accidental trauma with the majority of expertise and experience gained in the automotive industry.

The application of these principles to battlefield injury is discussed in this issue. First, detailed analysis of the injury patterns allows development of a biomechanical hypothesis. The relevance of such epidemiological papers must be maintained by ensuring sound methodology, as discussed in this issue by Gupta *et al*.^11^ Although analysis of clinical data may suggest the attributable mechanism, some form of experimental validation is invariably required.

Explosions are inherently chaotic and the recreation of blast conditions in a laboratory setting requires a degree of ingenuity and the controlled separation of the blast effects. Experiments should be reproducible, consistent and comparable. Guidelines for the recreation of these conditions (as described by Josey *et al* for primary blast exposure in this issue) are essential so that the dose of blast (be it overpressure, fragmentation or high rate blunt loading) be relevant and readily replicated.^12^

Measurement of the injurious response requires the use of an appropriate model. While postmortem human surrogates provide the greatest biomechanical fidelity for human injury, they have significant limitations for the replication of injuries to soft tissues, internal organs and for the measurement of physiological and cellular responses. Instead, an integrated research strategy must include *in vitro*, *in vivo*, *in silcio* and *ex vivo* models. Similar guidelines should be applied across each of research domains. The article by Watts *et al* in this issue suggests appropriate guidelines for the use of animals in order align blast research with the ‘3 Rs’ of animal work: replacement, reduction and refinement.^13^ Application of advanced technologies allow the translation of basic science and in vivo models into high fidelity computational simulations which reduce the need for expensive and logistically difficult physical experiments. The development of a computational model from in vivo data is demonstrated by Haque *et al* in this issue.^14^

Each of these steps is essential for understanding of the relationship between the blast ‘dose’ and the injury ‘response’. The capability of equipment designers and engineers to protect against a specified threat (as described by Sedman *et al* in this issue) is dependent on this process.^15^

This research chain requires expertise in different areas. Engineers, biologists, physicists and computer scientists all have important roles to play in an integrated research strategy. In this issue, Nguyen *et al* show the diverse experimental capabilities made possible by this form of academic cooperation.^16^ Successful collaboration requires the forging of a shared mission and the development of ‘T-shaped’ researchers who are able to cultivate their own discipline and look beyond it.^17^

Medical involvement is important in the blast injury research domain. Although the research role of the clinician has classically been to determine the optimal clinical care required for patients, clinicians are among those best placed to examine injury and understand the importance of injury mitigation to eventual outcome. The ‘surgeon-scientist’ is well established in clinical and preclinical research, but injury prevention and survivability research perhaps requires the ‘surgeon-engineer’ with an in-depth understanding of injury mechanism and quantification.

This role requires a change in perspective on trauma care. Opportunities of the clinician to influence survival should not begin at the point of injury but extend across the full spectrum to consider the cause and context of injury. The ‘Left of Bang’ approach to trauma has been eloquently discussed by Eisenstein *et al* 18 with particular regard to physiological pretrauma intervention.^19^ Although the translation of this concept to blast injury protection is apparent, Left of Bang should be considered by all trauma interested clinicians, military and civilian, who are well placed to identify and instigate those protective, behavioural, social and political changes which will reduce or mitigate injury.
